# Differential radiological features of patients infected or colonised with slow-growing non-tuberculous mycobacteria

**DOI:** 10.1038/s41598-024-64029-0

**Published:** 2024-06-10

**Authors:** Teodora Biciusca, Ann-Sophie Zielbauer, Thomas Anton, Lisa Marschall, Raja Idris, Julia Koepsell, Lisa J. Juergens, Jennifer Gotta, Vitali Koch, Thomas A. Wichelhaus, Thomas J. Vogl, Maria J. G. T. Vehreschild, Simon S. Martin, Nils Wetzstein

**Affiliations:** 1 Department of Radiology, Goethe University, University Hospital Frankfurt, Frankfurt am Main, Germany; 2 Department of Internal Medicine, Infectious Diseases, Goethe University, University Hospital Frankfurt, Theodor-Stern-Kai 7, 60590 Frankfurt, Germany; 3 Institute of Medical Microbiology and Infection Control, Goethe University, University Hospital Frankfurt, Frankfurt am Main, Germany; 4 Fraunhofer Institute for Translational Medicine and Pharmacology (ITMP), Frankfurt am Main, Germany

**Keywords:** NTM, Non-tuberculous mycobacteria, *Mycobacterium avium* complex, MAC, *Mycobacterium simiae*, *Mycobacterium kansasii*, *Mycobacterium xenopi*, Medical research, Infectious-disease diagnostics

## Abstract

Non-tuberculous mycobacterial pulmonary disease (NTM-PD) is considered a growing health concern. The majority of NTM-PD cases in Europe are caused by slow-growing mycobacteria (SGM). However, distinct radiological features of different SGM remain largely uninvestigated. We applied a previously described radiological score to a patient cohort consisting of individuals with isolation of different SGM. Correlations between clinical data, species and computed tomography (CT) features were examined by logistic and linear regression analyses, as well as over the course of time. Overall, 135 pulmonary CT scans from 84 patients were included. The isolated NTM-species were mainly *Mycobacterium avium* complex (MAC, n = 49), as well as 35 patients with non-MAC-species. Patients with isolation of *M. intracellulare* had more extensive CT findings compared to all other SGM species (coefficient 3.53, 95% Cl − 0.37 to 7.52, p = 0.075) while patients meeting the ATS criteria and not undergoing therapy exhibited an increase in CT scores over time. This study provides insights into differential radiological features of slow-growing NTM. While *M. intracellulare* exhibited a tendency towards higher overall CT scores, the radiological features were similar across different SGM. The applied CT score might be a useful instrument for monitoring patients and could help to guide antimycobacterial therapy.

## Introduction

Infections due to non-tuberculous mycobacteria (NTM) are considered an increasing health concern in countries with low tuberculosis (TB) incidence^1^. *Mycobacterium avium* complex (MAC) is the most frequent species group isolated in Europe and comprises *M. avium, M. intracellulare,* and *M. chimaera* among others^2^. Besides the MAC, other slow-growing NTM, such as *M. kansasii*, *M. xenopi*, *M. simiae*, can cause disease^3,4^.

Non-tuberculous mycobacterial pulmonary disease (NTM-PD) represents the primary clinical manifestation associated with NTM infections^3,5^. Chronic pre-existing pulmonary conditions, such as chronic obstructive pulmonary disease (COPD), asthma, and bronchiectasis, stand as the principal predisposing factors for the onset of NTM-PD^6–8^.

NTM-PD lesions display notable radiological heterogeneity, encompassing a spectrum of characteristics such as nodular infiltrates, bronchiectasis, consolidations, and cavities^3^. Ascertaining specific lesions as attributable to NTM can prove challenging, as most patients have pre-existing chronic lung conditions. The radiological manifestations of NTM lung disease can be broadly classified into two principal forms: the fibrocavitary and nodular-bronchiectatic form^3,9^. Previous research has demonstrated that patients with NTM infections lacking cavities but presenting with nodular bronchiectatic changes generally have a more positive prognosis and show an improved response to therapy compared to those with cavities^10,11^. Moreover, the presence of consolidations is also associated with a less favorable prognosis^12^.

To objectively evaluate the severity of radiological changes, Song et al. developed a scoring system specifically for MAC lung infections^13^. This scoring system, inspired by a previously proposed system by Helbich et al. for cystic fibrosis^14^, assigns points based on the severity, prevalence, and size of each radiological change associated with MAC lung involvement. Except for this species group, there is a scarcity of radiographic investigations focusing on distinct NTM species and, to our knowledge, the described score has not yet been applied outside the MAC^15–17^.

As the optimal treatment regimens differ, establishing a species-specific diagnosis is needed^18^. Therefore, this study sought to examine the computed tomography (CT) features of various slow-growing NTM species using the standardized and objective radiological scoring system proposed by Song et al.^13^. Our objective was to explore the correlation between radiological scores and the clinical impact of NTM-isolation, as well as to identify potential differences between MAC and other slow-growing NTM species.

## Methods

### Patient inclusion and clinical parameters

All patients with pulmonary isolation of SGM treated at University Hospital Frankfurt from 2006 to 2021 and available CT-scans of the lung were included in the study. Patients with cystic fibrosis (CF) or human immunodeficiency virus (HIV) infection as comorbidities were excluded. Clinical data, including gender, country of origin, age, other relevant comorbidities, the administration of immunosuppressive therapies, death, clinical symptoms and manifestations, as well as the administered antimycobacterial therapy, were extracted by three investigators through chart review (ASZ, TA, LM). NTM-PD was defined as fulfillment of the ATS criteria⁠^3,18^. Microbiological data, including the mycobacterial species, genotypic and phenotypic drug resistance, were recorded. The MAC species was determined using the GenoType NTM-DR (HAIN Life Sciences, Nehren, Germany). NTM species other than MAC were determined by line probe assay (GenoType Common Mycobacteria (CM), Hain Life Sciences, Nehren, Germany) and internal transcribed spacer polymerase chain reaction (ITS-PCR), respectively.

### Radiological assessment and CT imaging parameters

High resolution computer tomography (HRCT) scans were performed as part of patient routine care at the University Hospital Frankfurt using a Siemens Somatom Definition AS CT scanner. Within the examinations, native and portal venous sequences were performed. Contraindications for a CT examination with contrast agent included iodine allergy and renal insufficiency or hyperthyroidism.

Two different radiologists (TB, SM) reviewed HRCT images, quantifying lung involvement using a specialized radiological score proposed by Song et al. for MAC lung disease^13^. This scoring system allocates a maximum total score of 42 to assess the overall extent of a lung lesion (minimum score 0, Supplementary Table 1). The score was assigned based on factors such as lobar volume decrease, the presence, severity, and extent of various abnormalities including bronchiectasis, bronchiolitis, cavity, nodules, consolidation, bullae, emphysema, and mosaic perfusion in both lungs. Bronchiectasis was identified when the bronchial lumen diameter exceeded that of the adjacent pulmonary artery without tapering. Bronchial wall thickening and mucus plugging were considered under bronchiectasis, with wall thickness measured as the ratio of airway wall thickness to the outer diameter of the corresponding bronchus. Bronchiolitis was defined by the presence of centrilobular small nodules and branching nodular structures (tree-in-bud signs) on HRCT scans. Definitions for bullae and emphysema adhered to the Fleischner Society's glossary of terms and mosaic perfusion was characterized by areas of decreased lung attenuation with oligemic pulmonary vessels. To assess the progression of lung involvement, if applicable, overall scores at HRCT baseline and follow-up ranging from 6 to 18 months later were aligned. All radiologists remained unaware of the patients' treatment status, allowing an unbiased estimation of the difference in radiological scoring values.

### Statistical analysis

All analyses and data visualizations were performed in R (version 4.3.1) using packages from the *tidyverse*^19,20^. Categorical variables are presented as numerator with denominator and percentages, while continuous variables are presented as median with interquartile range for non-normally distributed data and as mean with range for normally distributed data. Normality was assessed using the Shapiro–Wilk test. Univariate logistic regression was conducted to examine the association of mycobacterial species with the presence of different items of the score, using the *finalfit* package in R^21^. Finally, the association of mycobacterial species and overall score severity, as well as severity of single items expressed by numeric values (Supplementary Table S1) was assessed with a linear regression model with the same package and results reported as coefficients with 95% confidence interval (95% CI) and p-values. This analysis included only baseline CT images (one per patient). The Wilcoxon signed-rank test was used to identify statistically significant differences in the total CT score between baseline and follow-up CT scans. A significance level of alpha = 0.05 was used for all statistical tests.

### Ethical approval and consent to participate

The ethics committee of the Goethe University Hospital Frankfurt approved the study protocol (ref: 2022-162) and the study was carried out in accordance with the Declaration of Helsinki. As this is a retrospective study and all data was collected as part of standard care, no written consent was needed.

## Results

### Baseline characteristics and clinical manifestations

Overall, 135 pulmonary CT scans from 84 patients were included. Among them, 53 patients were male (63.1%) and 31 were female (36.9%) (Table 1). The median age was 64 years (IQR 55.75–73.0 years). A majority of these patients (50/84, 59.5%) had an underlying structural lung disease. Additionally, 33 patients suffered from malignancy (39.3%), 11 suffered from rheumatic disease (13.1%), and 40 were smokers (47.6%). Twenty-six patients received immunosuppressive therapy (31.0%) with prednisone being the most frequently administered substance (n = 25, 29.8%). The majority of patients were German born (77.4%). During the observation period, 17 of the patients deceased (20.2%).

**Table 1:** Baseline characteristics.

	All patients	ATS positive	ATS negative
	n = 84	n = 33	n = 51
	n/N (%)	n/N (%)	n/N (%)
Gender			
Male	53/84 (63.1%)	15/33 (45.5%)	38/51 (74.5%)
Female	31/84 (36.9%)	18/33 (54.5%	13/51 (25.5%)
Country of origin			
Germany	65/84 (77.4%)	26/33 (78.8%)	39/51 (76.5%)
Non-German-born	19/84 (22.6%)	7/33 (21.2%)	11/51 (21.6%)
Comorbidities			
Structural lung disease	50/84 (59.5%)	25/33 (75.8%)	25/51 (49.0%)
Malignancy	33/84 (39.3%)	8/33 (24.2%)	25/51 (49.0%)
Rheumatic disease	11/84 (13.1%)	7/33 (21.2%)	4/51 (7.8%)
Smoker	40/84 (47.6%)	17/33 (51.5%)	23/51 (45.1%)
Immunosuppressive			
Prednisone	25/84 (29.8%)	14/33 (42.4%)	11/51 (21.6%)
Other^a^	8/84 (9.5%)	2/33 (6.1%)	8/51 (15.7%)
Clinical symptoms			
Cough	46/84 (54.8%)	26/33 (78.8%	20/51 (39.2%)
Dyspnea	35/84 (41.7%)	14/33 (42.4%)	21/51 (41.2%)
Hemoptysis	14/84 (16.7%)	4/33 (12.1%)	10/51 (19.6%)
Lymphadenopathy	25/84 (29.8%)	8/33 (24.2%)	17/51 (33.3%)
Weight loss	27/84 (32.1%)	17/33 (51.5%)	10/51 (19.6%)
Fever	18/84 (21.4%)	11/33 (33.3%)	7/51 (13.7%)
Night sweat	10/84 (11.9%)	3/33 (9.1%)	7/51 (13.7%)
Mycobacterial species			
* M. avium*	27/84 (32.1%)	11/33 (33.3%)	16/51 (31.4%)
* M. xenopi*	16/84 (19.0%)	4/33 (12.1%)	12/51 (23.5%)
* M. kansasii*	12/84 (14.3%)	5/33 (15.2%)	7/51 (13.7%)
* M. chimaera*	11/84 (13.1%)	3/33 (9.1%)	7/51 (13.7%)
* M. intracellulare*	11/84 (13.1%)	9/33 (27.3%)	2/51 (3.9%)
* M. simiae*	6/84 (7.1%)	0/33 (0.0%)	6/51 (11.8%)
* M. malmoense*	1/84 (1.2%)	0/33 (0.0%)	1/51 (2.0%)
Outcome			
Deceased	17/84 (20.2%)	6/33 (18.2%)	11/51 (21.6%)

*ATS* American Thoracic Society.

^a^*MMF* Mycophenolate mofetil, *CSA* Cyclosporine A, *MTX* Methotrexate, *TNF* Tumor necrosis factor.

Thirty-three patients met the ATS criteria for NTM pulmonary disease (39.3%, Supplementary Table S2). This group comprised 13 patients classified as fibrocavitary, 11 as nodular-bronchiectactic, and nine as an unclassifiable disease type (39.4%, 33.3%, and 27.3%, respectively).

### Microbiological examinations, species distribution and antimycobacterial therapy

In 49 patients, MAC species were isolated by culture or identified by molecular methods (27 cases of *M. avium*, 11 of *M.* *intracellulare*, and 11 of *M. chimaera*). In addition, 16 patients with identification of *M. xenopi*, 12 with *M. kansasii*, six with *M. simiae* and one with *M. malmoense* were included (totaling 35 patients with isolation of non-MAC species) (Table 1). Eighty-two patients were culture-positive, 23 patients were positive in microscopy, 22 had positive PCR results, and 45 patients exhibited histological findings consistent with mycobacterial disease. 36.9% of patients received antimycobacterial therapy, with the majority of them (28/31, 90.0%) receiving regimens that included a macrolide, a rifamycin and ethambutol (Supplementary Table S3). In our cohort, susceptibility testing was performed in 25 cases, while genotypic and phenotypic drug resistance to macrolides and aminoglycosides was rare, with only three and two cases, respectively.

### Baseline CT score

**Figure 1:**
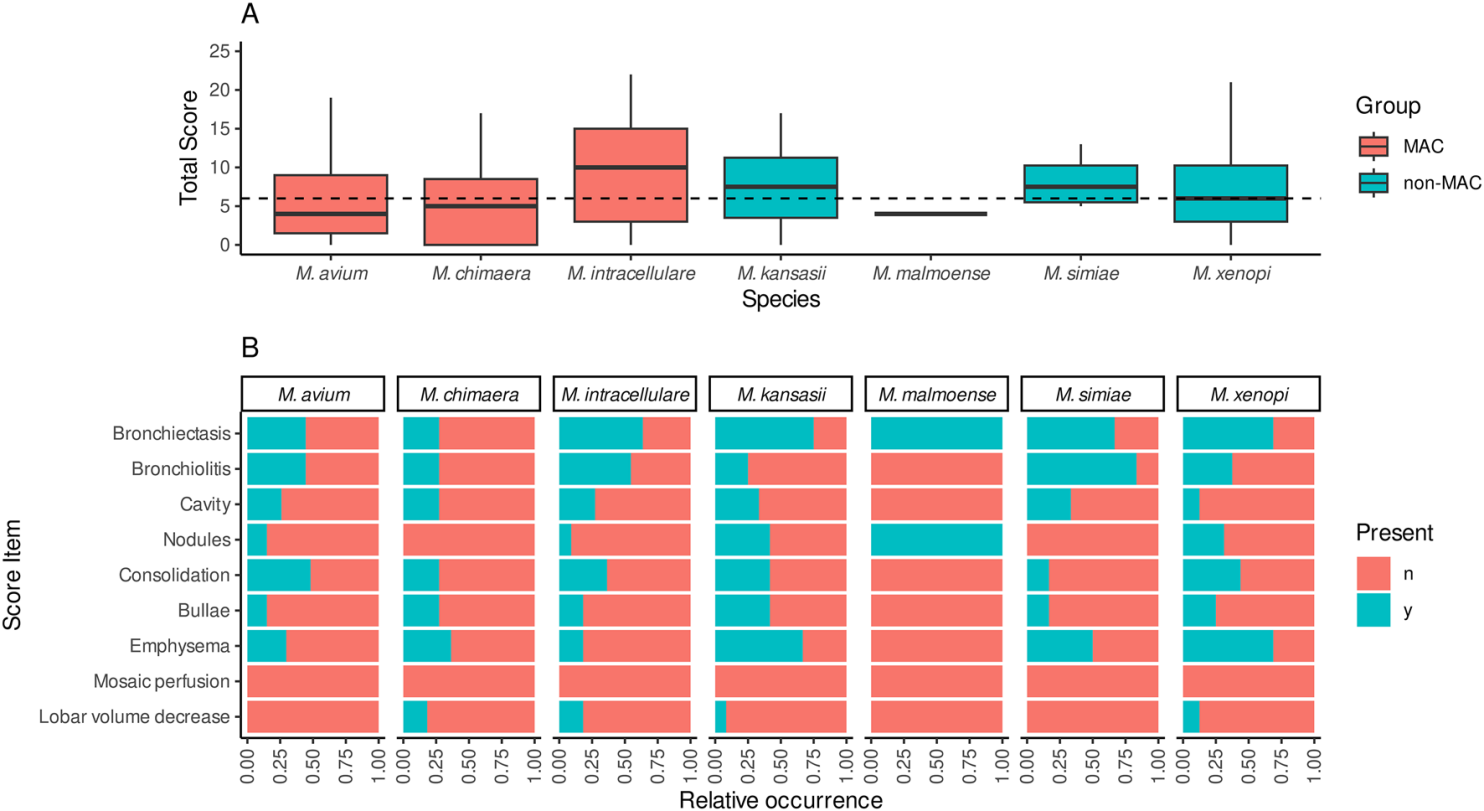
Baseline CT score for different NTM-species (**A**, dashed line marks the overall median score) and presence of single components of the applied CT score (**B**).

Baseline CT scans from 84 patients revealed a median CT score of 6 (IQR 3–10.25) (Fig. 1A, Table 2). Specifically, the CT score was 5 (IQR 1–10) for MAC species and 7 (IQR 4–11) for non-MAC species (p = 0.08). *M. intracellulare* displayed the highest median CT scores when compared to other MAC members (10 vs. 4.5, p = 0.17), as well as when compared to all other SGM-species (linear regression coefficient 3.53, 95% Cl − 0.37 to 7.52, p = 0.075). Patients who fulfilled the ATS criteria exhibited significantly higher CT scores (coefficient 3.26, 95% Cl 0.9–5.62, p = 0.007). In patients with *M. intracellulare* isolation, CT scores were significantly higher in those with fulfillment of the ATS criteria, than in those without (11 vs. 0.5, p = 0.04, Wilcoxon signed-rank test).

**Table 2:** Overall CT-scores differentiated by species, fulfillment of the ATS criteria, and time point of CT-scan.

	All patients		ATS positive		ATS negative	
	n	median score, IQR	n	median score, IQR	n	median score, IQR
Baseline CT						
All species	84	6 (3–10.25)	33	9 (4–11)	51	5 (1–8)
* M. avium*	27	4 (1.5–9)	13	7 (4–10)	14	2.5 (0.25–6.25)
* M. intracellulare*	11	10 (3–15.0)	9	11 (4–16)	2	0.5 (0.25–0.75)
* M. chimaera*	11	5 (0–8.5)	3	10 (6.5–13.5)	8	2.5 (0–6.25)
* M. kansasii*	12	7.5 (3.5–11.25)	5	9 (7–11)	7	7 (2.5–0.75)
* M. xenopi*	16	6 (3–10.25)	3	14 (7.5–17.5)	13	6 (3–9)
* M. simiae*	6	7.5 (3.5–11.25)	0	NA	6	7.5 (5.5–10.25)
* M. malmoense*	1	4	0	NA	1	4
Follow-up CT						
All species	51	6 (3.5–11.5)	25	7 (4–12)	26	7 (4–12)
* M. avium*	17	5 (1–7)	12	6 (3.75–7)	5	0 (0–5)
* M. intracellulare*	9	15 (4–17)	6	16.5 (15.25–17.75)	3	4 (3.5–8)
* M. chimaera*	7	5 (0–8.5)	3	5 (2.5–12)	4	3.5 (0–7.75)
* M. kansasii*	6	8.5 (4.5–11.75)	3	11 (6.5–11.5)	3	6 (5–15)
* M. xenopi*	8	8 (4.75–12.25)	1	12	7	7 (4.511)
* M. simiae*	1	11 (7.5)	0	NA	3	11 (7.5–13)
* M. malmoense*	1	3	0	NA	1	3

*CT* computed tomography, *IQR* interquartile range, *ATS* American Thoracic society, *NA* not applicable.

**Figure 2:**
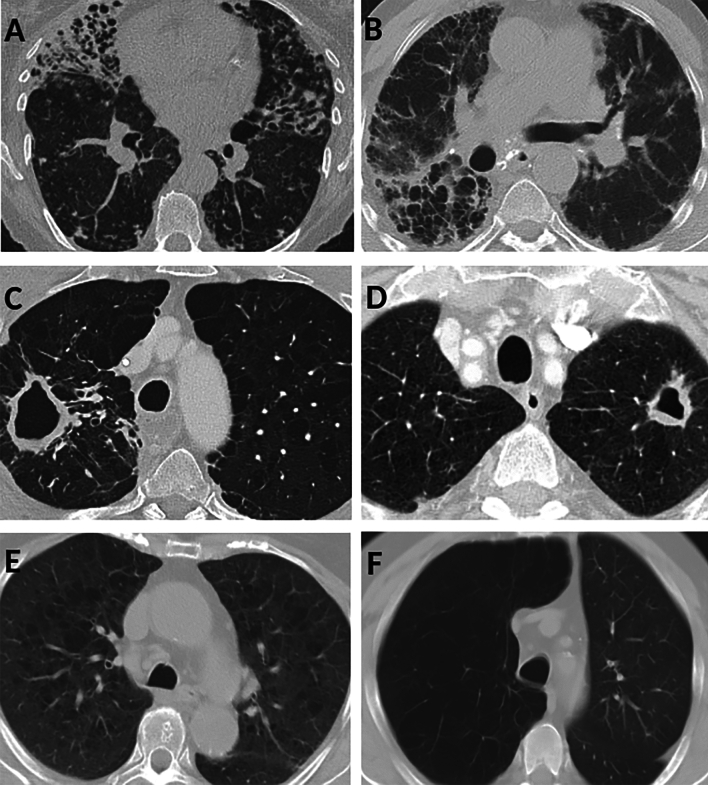
Exemplary CT images of included patients with SGM: high grade bronchiectasis in two patients with *M. intracellulare* (**A**,**B**), cavity and emphysema in a patient with *M. chimaera* (**C**) and with *M. avium* (**D**), emphysema in a patient with *M.* *xenopi* (**E**) and with *M. kansasii* (**F**).

Analyzing the individual components of the Score by Song et al., no patients exhibited mosaic perfusion and there were no significant differences in the prevalence of lobar volume reduction, bullae, consolidations, cavities, nodules and bronchiectasis across patients with the seven different SGM species included in the logistic regression model (Fig. 1B). However, in patients with pulmonary isolation of *M. intracellulare,* the severity of bronchiectasis was more pronounced (coefficient 3.01, 95% CI 0.65–5.38, p = 0.013). For patients with isolation of *M. kansasii* and *M. xenopi*, emphysema was more severe (coefficient 4.75, 95% Cl 1.16–22.44, p = 0.036 and coefficient 5.22, 95% Cl 1.43–21.61, p = 0.016, respectively). Exemplary CT scan images of the patients included in this study are provided in Fig. 2.

### Course of CT features over time

**Figure 3:**
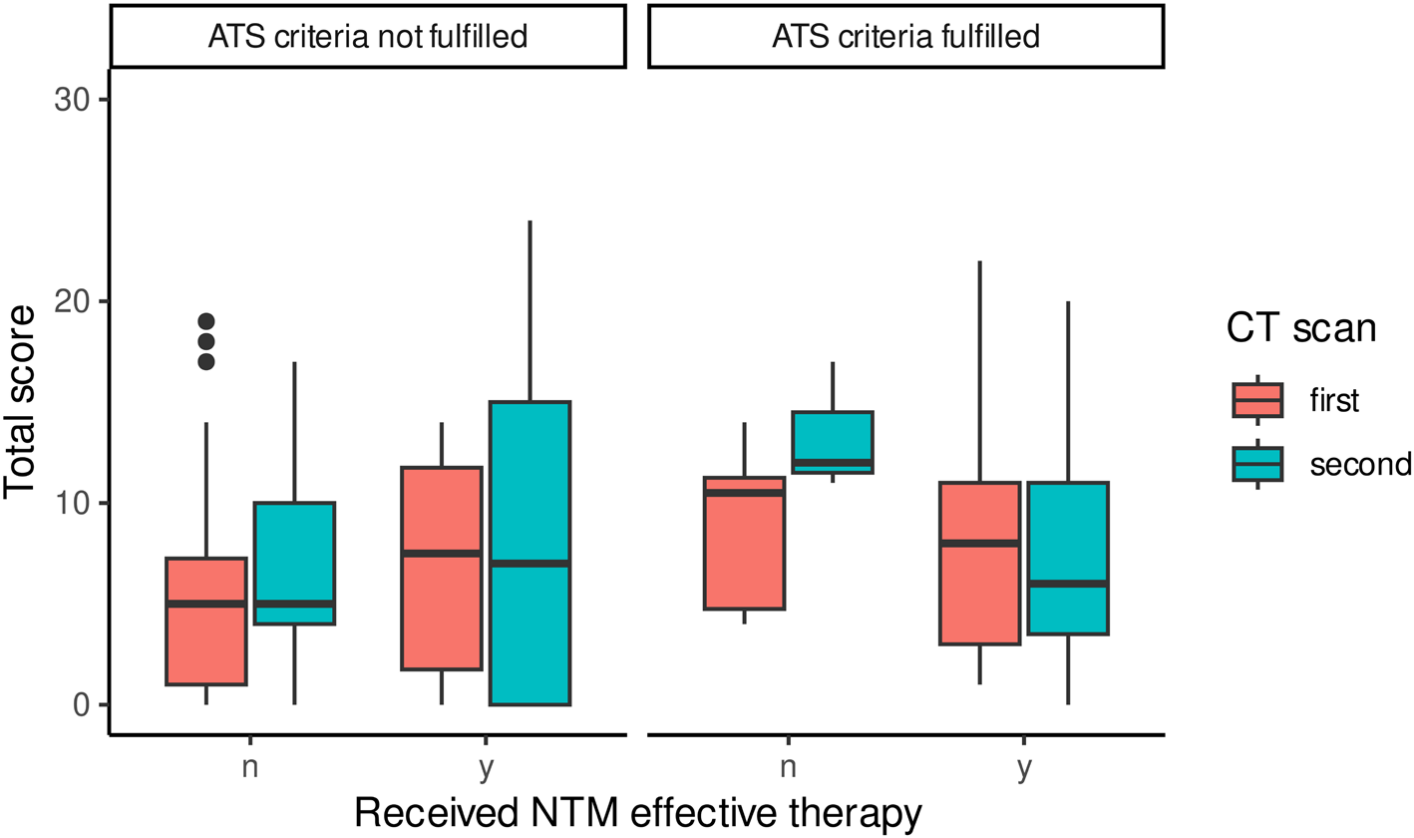
Temporal evolution of CT scores in patients with and without antimycobacterial therapy.

For 47 patients, at least one sequential CT scan was available. We observed an improvement in the overall CT score among patients receiving antimycobacterial therapy, regardless whether they met the ATS criteria or not (8 IQR 3–12 vs. 6.5 IQR 2.75–12.75, Fig. 3). However, this improvement was not statistically significant (p = 0.55). Conversely, patients who met the ATS criteria but did not receive NTM-effective therapy tended to experience a worsening of their overall CT score (10.5 IQR 4.75–11.25 vs. 12 IQR 11.5–14.5, p = 0.15), whereas in patients who did not meet the ATS criteria, the score remained largely unchanged (5.0 IQR 1–7.25 vs. 5.0 IQR 4–10, p = 0.46).

## Discussion

Our study implements the radiological score by Song et al. to evaluate a group of European patients who have been infected or colonised by various slow-growing NTM. We find only slight differences in the radiological features of different SGM species, but a general tendency towards higher scores in those with *M. intracellulare.* Further, we show an increase of score severity in ATS-positive patients that are not treated indicating a possible value of the score to guide the initiation of antimycobacterial therapy.

Among the 84 patients in our cohort, there is a noticeable trend suggesting that those with *M. intracellulare* isolation tend to exhibit higher CT scores. Moreover, patients with *M. intracellulare* showed notably more pronounced severity of bronchiectasis. This is in line with previous studies that demonstrated a higher proportion of patients with *M. intracellulare* with significantly higher frequencies and severity of bronchiectasis compared to other NTM species^15^. On the other hand, in previous studies, *M. chimaera* was found to be of lower clinical significance when isolated from pulmonary specimens, reflecting its comparatively lower virulence in comparison to other members of the MAC^22^. Different non-MAC species exhibited relatively similar CT scores. Overall, patients fulfilling the ATS criteria displayed higher CT scores, than those that did not, confirming previous studies: Garcia et al. have shown higher extent of bronchiectasis in patients with NTM-PD compared to those that are solely colonised by NTM^23^.

When analyzing the individual components of the score developed by Song et al., we observed that there were no significant disparities in the prevalence of lobar volume reduction, bullae, consolidations, cavities, and nodules among patients with isolation of different SGM species. Nevertheless, for patients with *M. kansasii* and *M. xenopi*, a significantly larger proportion exhibited emphysema, results which align with findings similar to those reported by Hollings et al.^15^. Previously, it has been observed that *M. kansasii* and *M. xenopi* are much more commonly associated with cavities or consolidations^12,22^. However, we could not detect significant differences in the extent of cavities between the different slow-growing species. This lack of discriminative power might be partially explained by our limited sample size. Additionally, although we excluded patients with HIV and CF as comorbidities, the included patient cohort might still be heterogeneous, potentially leading to further variations in the applied score. Consequently, achieving a specific differentiation between various slow-growing mycobacterial species based solely on radiological images appears challenging. Similar observations have recently been described in a study investigating the radiological characteristics of NTM-infection in patients with bronchiectasis^24^. Here, a concomitant *Pseudomonas aeruginosa* infection already resembled NTM radiographically, underlining the difficulties of pathogen prediction by radiological features alone. Finally, patients with NTM-PD often suffer from polymicrobial infections further complicating the identification of attributable radiological changes^25,26^.

In our temporal analysis, we observed largely stable overall scores in patients who did not fulfill the ATS criteria, regardless of whether they received therapy or not. Conversely, patients who did meet the ATS criteria showed a decrease in score with antimycobacterial therapy and an increase if left untreated, although these changes were not statistically significant. These results align with those by Kwak et al. who observed an increase in the severity and extent of bronchiectasis, and the extent of nodules on the onset of NTM-PD in a longitudinal observation^27^. Radiographic improvement may be challenging due to concomitant lung disease of the patients in our study. As expected, this data suggests that the CT score by Song et al. could potentially serve as a monitoring parameter in patients for whom a “watch-and-wait” strategy has been adopted and its temporal evolution could be used as an indicator for initiating therapy.

This study has several limitations. First, although we were able to include 84 patients and a total of 135 CT scans, the sample size for certain species remains limited. However, we deliberately chose not to combine different MAC or non-MAC species for statistical analyses. Second, as this is a retrospective study, follow-up CT scans were not performed at fixed time intervals and did not follow a standardized study protocol. In addition, the investigated study cohort might be pre-selected, as we included only patients with an available HRCT. Third, despite excluding HIV and CF patients from the analysis, the heterogeneity of the study cohort may still impact the interpretation of our results. Nevertheless, in our view, this study contributes valuable insights into the diagnostic challenges of NTM-PD.

## Conclusion

In conclusion, this study provides insights into the differential radiological features of different slow-growing NTM. Understanding the heterogeneity among NTM species, their clinical manifestations, and the importance of radiological assessments can help improve the diagnosis and treatment of NTM-PD. The applied CT score could be a useful tool for monitoring patients and its temporal evolution could guide antimycobacterial therapy.

### Supplementary Information

Supplementary Information.

## Data Availability

The datasets used and analyzed during the current study are available from the corresponding author upon reasonable request.

## Abbreviations

ATS: American thoracic society
CF: Cystic fibrosis
CM: Common mycobacteria
COPD: Chronic obstructive pulmonary disease
CT: Computer tomography
HIV: Human immunodeficiency virus
ITS-PCR: Internal transcribed spacer polymerase chain reaction
MAC: *Mycobacterium avium* complex
NTM: Non-tuberculous mycobacteria
NTM-PD: NTM pulmonary disease
OR: Odds ratio
PCR: Polymerase chain reaction
SGM: Slow-growing mycobacteria
TB: Tuberculosis
95% CI: 95% Confidence interval
