# Supplementary material for: Differential radiological features of patients infected or colonised with slow-growing non-tuberculous mycobacteria
Source: Sci Rep. 2024 Jun 10;14:13295. doi: 10.1038/s41598-024-64029-0 (PMC11164953; doi:10.1038/s41598-024-64029-0)
Supplement: Supplementary file 1 — Supplementary Information. [file 41598_2024_64029_MOESM1_ESM.docx]

**Supplementary tables**

Table S1: CT-Score developed by Song et al. for MAC lung infections.

| **CT findings**  **(maximum score)** | **Score** | | | |
| --- | --- | --- | --- | --- |
|  | **0** | **1** | **2** | **3** |
| **Bronchiectasis (12 points)** | | | | |
| **Severity** | Absent | Mild (bronchus diameter larger than a vessel diameter). | Moderate (bronchus diameter 2-3 times larger than a vessel diameter). | Severe (bronchus diameter 3 times larger than a vessel diameter). |
| **Extent** |  | 1-5 segments | 6-9 segments | > 9 segments |
| **Bronchial wall thickening** |  | Mild (bronchus wall larger than a vessel diameter). | Moderate (bronchus wall 2-3 times larger than a vessel diameter). | Severe (bronchus wall 3 times larger than a vessel diameter). |
| **Mucus plugging** |  | 1-5 segments | 6-9 segments | > 9 segments |
| **Bronchiolitis (6 points)** | | | | |
| **Severity** | Absent | Mild (peripheral lung, <2cm from pleura). | Moderate (>2cm from pleura) | Severe (spread to the central lung) |
| **Extent** |  | 1-5 segments | 6-9 segments | > 9 segments |
| **Cavity (6 points)** | | | | |
| **Severity** | Absent | Mild (diameter  < 3 cm) | Moderate (diameter = 3-5 cm) | Severe (diameter > 5 cm) |
| **Extent** |  | 1-3 in number | 3-5 in number | > 5 in number |
| **Other score items** | | | | |
| **Nodules**  **(10-30 mm in diameter)**  **(3 points)** | Absent | 1-5 segments | 6-9 segments | > 9 segments |
| **Consolidations, lobular, segmental or peribronchial**  **(3 points)** | Absent | < 3 segments | 3-5 segments | > 5 segments |
| **Bullae**  **(3 points)** | Absent | Unilateral (< 4 in number) | Bilateral (< 4 in number) | Bilateral (> 4 in number) |
| **Emphysema**  **(3 points)** | Absent | - 1. segments | > 5 segments | - |
| **Mosaic perfusion**  **(3 points)** | Absent | - 1. segments | > 5 segments | - |
| **Lobar volume decrease**  **(3 points)** | Absent | 1 lobe | 2 lobes | > 3 lobes |

Table S2: Fulfillment of the ATS Criteria.

|  |  | **n/N (%)** |
| --- | --- | --- |
| **ATS criteria positive** |  | 33/84 (39.3%) |
|  | Pulmonary Symptoms and appropriate radiology | 48/84 (57.1%) |
|  | Exclusion of other diagnosis | 36/84 (42.9%) |
|  | Two positive sputa | 21/84 (25.0%) |
|  | Positive BAL | 59/84 (70.2%) |
|  | Positive bronchial biopsy | 13/84 (15.5%) |
| **Disease type** |  |  |
|  | Fibrocavitary | 13/33 (39.4%) |
|  | Nodular bronchiectactic | 11/33 (33.3%) |
|  | Other | 9/33 (27.3%) |

ATS: American Thoracic Society

Table S3: Microbiological characteristics of included patients

|  |  |  | n/N (%) |
| --- | --- | --- | --- |
| Mycobacterial species | |  |  |
|  | *M. avium* |  | 27/84 (32.1%) |
|  | *M. xenopi* |  | 16/84 (19.0%) |
|  | *M. kansasii* | | 12/84 (14.3%) |
|  | *M. chimaera* |  | 11/84 (13.1%) |
|  | *M. intracellulare* |  | 11/84 (13.1%) |
|  | *M. simiae* | | 6/84 (7.1%) |
|  | *M. malmoense* |  | 1/84 (1.2%) |
| Genotypic drug resistance | |  |  |
|  | Macrolide resistant | | 3/25 (12.0%) |
|  | Aminoglycoside resistant | | 2/25 (8.0%) |
| Phenotypic drug resistance | |  |  |
|  | Macrolide resistant | | 3/25 (12.0%) |
|  | Aminoglycoside resistant | | 2/25 (8.0%) |
| Received NTM Therapy | |  | 31(84 (36.9%) |
| Macrolide |  |  | 28/31 (90.0%) |
|  | Azithromycin | | 8/28 (28.6%) |
|  | Clarithromycin | | 15/28 (53.6%) |
| Ethambutol |  |  | 28/31 (90.0%) |
| Rifamycin |  |  | 28/31 (90.0%) |
|  | Rifabutin |  | 15/28 (53.6%) |
|  | Rifampicin |  | 13/28 (46.4%) |
| Fluoroquinolones | |  | 10/31 (32.3%) |
|  | Levofloxacin |  | 2/10 (20.0%) |
|  | Moxifloxacin |  | 6/10 (60.0%) |
| Amikacin |  |  | 8/31 (25.8%) |
| Linezolid |  |  | 1/31 (3.2%) |
| Isoniazid |  |  | 10/31 (32.3%) |
| Pyrazinamide | |  | 9/31 (29.0%) |
| Clofazimine |  |  | 2/31 (6.5%) |
| Others |  |  | 5/31 (16.1%) |

M.: myocobacteria; NTM: non-tuberculous mycobacteria
